# REG1A and RUNX3 Are Potential Biomarkers for Predicting the Risk of Diabetic Kidney Disease

**DOI:** 10.3389/fendo.2022.935796

**Published:** 2022-07-22

**Authors:** Xinyu Wang, Han Wu, Guangyan Yang, Jiaqing Xiang, Lijiao Xiong, Li Zhao, Tingfeng Liao, Xinyue Zhao, Lin Kang, Shu Yang, Zhen Liang

**Affiliations:** ^1^ Department of Geriatrics, The Second Clinical Medical College of Jinan University, Shenzhen People’s Hospital, Shenzhen, China; ^2^ Department of Endocrinology, The Second Clinical Medical College of Jinan University, Shenzhen People’s Hospital, Shenzhen, China; ^3^ Department of Health Management, The Second Clinical Medical College of Jinan University, Shenzhen People’s Hospital, Shenzhen, China; ^4^ Department of Nephrology, The Second Clinical Medical College of Jinan University, Shenzhen People’s Hospital, Shenzhen, China; ^5^ The Biobank of National Innovation Center for Advanced Medical Devices, Shenzhen People’s Hospital, Shenzhen, China; ^6^ Shenzhen Clinical Research Center for Aging, Shenzhen, China

**Keywords:** diabetic kidney disease, biomarkers, diagnosis, gene expression omnibus, disease risk prediction

## Abstract

Diabetic kidney disease (DKD) is the leading cause of end-stage renal disease. Clinical features are traditionally used to predict DKD, yet with low diagnostic efficacy. Most of the recent biomarkers used to predict DKD are based on transcriptomics and metabolomics; however, they also should be used in combination with many other predictive indicators. The purpose of this study was thus to identify a simplified class of blood biomarkers capable of predicting the risk of developing DKD. The Gene Expression Omnibus database was screened for DKD biomarkers, and differentially expressed genes (DEGs) in human blood and kidney were identified *via* gene expression analysis and the Least Absolute Shrinkage and Selection Operator regression. A comparison of the area under the curve (AUC) profiles on multiple receiver operating characteristic curves of the DEGs in DKD and other renal diseases revealed that *REG1A* and *RUNX3* had the highest specificity for DKD diagnosis. The AUCs of the combined expression of *REG1A* and *RUNX3* in kidney (AUC = 0.929) and blood samples (AUC = 0.917) of DKD patients were similar to each other. The AUC of blood samples from DKD patients and healthy individuals obtained for external validation further demonstrated that *REG1A* combined with *RUNX3* had significant diagnostic efficacy (AUC=0.948). *REG1A* and *RUNX3* expression levels were found to be positively and negatively correlated with urinary albumin creatinine ratio and estimated glomerular filtration rate, respectively. Kaplan-Meier curves also revealed the potential of *REG1A* and *RUNX3* for predicting the risk of DKD. In conclusion, *REG1A* and *RUNX3* may serve as biomarkers for predicting the risk of developing DKD.

## Introduction

With the increasing incidence of diabetes mellitus (DM) worldwide, diabetic kidney disease (DKD) has become the leading cause of end-stage renal disease with a high mortality rate (1). Approximately one-quarter of people with DM end up developing DKD (2). DKD diagnosis is primarily based on renal pathology and/or clinical manifestations and involves the determination of glomerular filtration rate (eGFR) and urinary protein levels in addition to clinical features such as the duration of DM and the presence of diabetic retinopathy (3, 4). However, eGFR and albuminuria are deficient in terms of sensitivity and specificity, while renal puncture is not widely available due to its invasiveness. Therefore, markers associated with DKD risk need to be identified for successful diagnosis and prevention. A meta-analysis showed that urinary transferrin excretion rate was a good predictor of DKD occurrence (5). Kidney injury molecule 1 is a membrane protein expressed in the apical membrane of proximal tubule cells and is of great value in assessing the progression of DKD (6). After 12 years of follow-up in a cohort study of 628 patients with DM, plasma tumor necrosis factor levels were found to be associated with early decline in eGFR was strongly correlated (7). Older age, male sex, prolonged diabetes duration, hypertension, glycated hemoglobin levels (HbA_1c_), and plasma triglyceride levels were identified as risk factors for proteinuria in patients with type 2 DM in a previous meta-analysis of 13 studies (8). Several biomarkers to assess DKD risk have been identified as a result of recent advancements in analytical techniques as well. Mayer et al. also identified nine serum markers associated with decreased GFR in Type 2 DM, including chitinase 3-like protein 1, growth hormone 1, hepatocyte growth factor, matrix metalloproteinase 2 (MMP2), MMP7, MMP8, MMP13, tyrosine kinase, and tumor necrosis factor receptor 1 (9). The surrogate markers for micro- and macro-vascular hard endpoints for innovative diabetes tools study further identified five serum markers (fibroblast growth factor 21, symmetric-to-asymmetric dimethylarginine ratio, β2-microglobulin, C16-acylcarnitine, and kidney injury molecule 1) with significant prediction potential for GFR decline in patients with type 2 DM (10). Although traditional clinical characteristics are easily determined, their prognostic value is limited. In general, serum biomarkers have a higher predictive efficacy than clinical characteristics; however, multiple biomarkers should be used in combination to achieve such efficacy. Simple biomarker combinations often lack specificity. Thus, widespread application of serum biomarkers is hindered.

**Figure 1:**
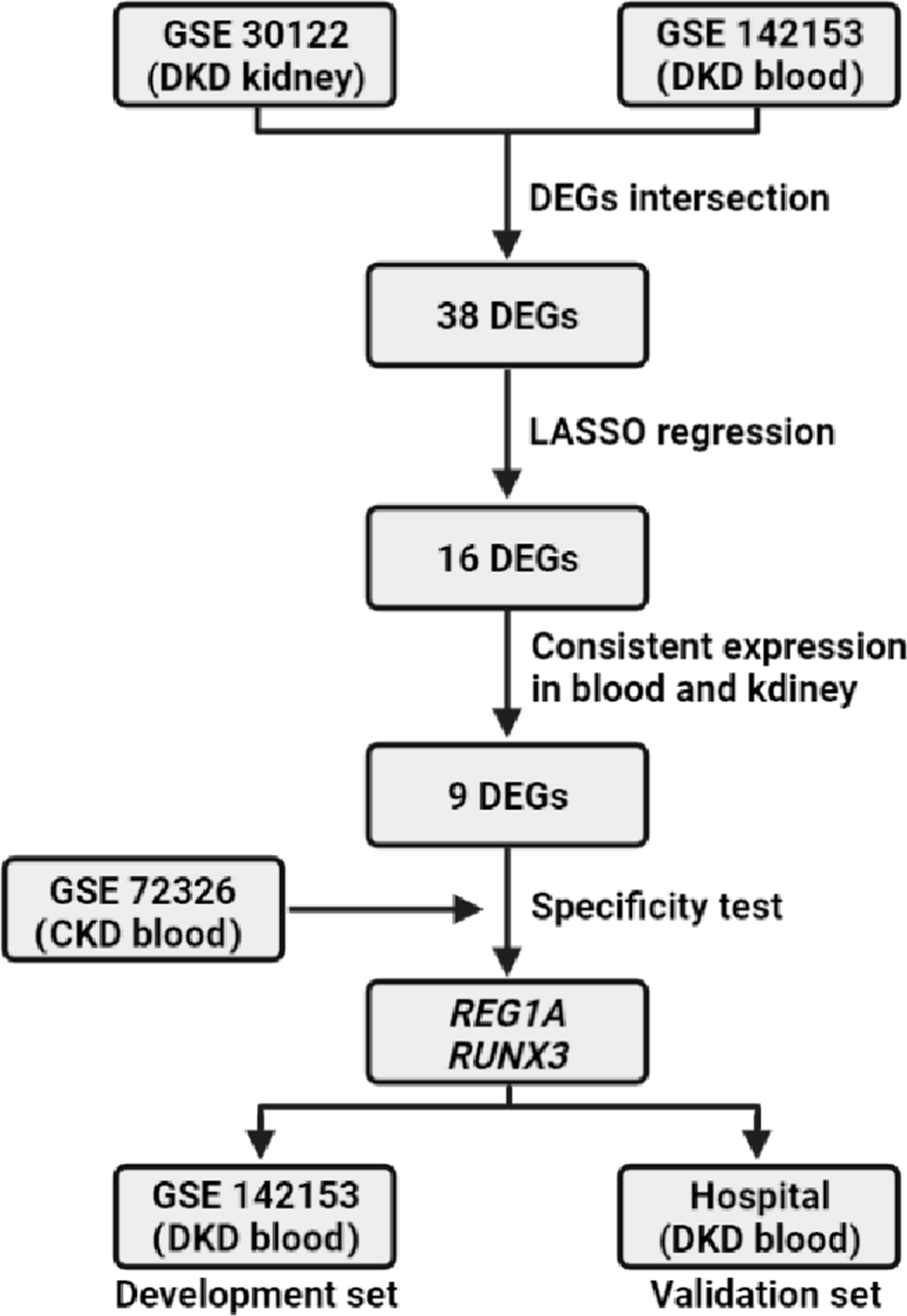
Flowchart. DKD, diabetic kidney disease; CKD, Chronic kidney disease.

Genome-wide transcriptome analysis has been widely used in the field of DKD to help gain insight into disease pathogenesis, molecular classification and identification of biomarkers (11). These gene expression matrices are included in the Gene Expression Omnibus (GEO) database to facilitate integration and reanalysis by researchers. The goal of this study was to establish a simplified class of blood biomarkers for predicting the risk of DKD. The study flow is illustrated in **Figure 1**.

## Method

### Data Acquisition

We acquired gene expression profile datasets GSE30122 (11), GSE142153 (12), and GSE72326 (13) from the GEO database (14) (https://www.ncbi.nlm.nih.gov/geo/), and performed gene ID and symbol conversions *via* Perl scripts (15). GSE30122 included kidney tissue from patients with pathologically confirmed DKD stage 4-5, with healthy kidney tissue as a control. GSE142153 incorporates peripheral blood from patients with a clinical diagnosis of DKD stages 3-5, with healthy human blood as a control. GSE142153 included peripheral blood from patients with confirmed CKD, with blood from people without kidney disease as a control.

### Identification of Differentially Expressed Genes

R software (version 4.1.0, http://r-project.org/) was used for data analysis and plotting. The “limma” R package (16) was used to screen for DEGs, and heatmap and volcano plots of DEGs were constructed by the “ggplot2” package (17) to visualize the expression levels of DEGs. p < 0.05 was considered statistically significant.

### Screening and Validation of Diagnostic Markers

The Least Absolute Shrinkage and Selection Operator (LASSO) method, as implemented in the “glmnet” software package, was used for reduction of data dimensionality and selection of predictive features for DKD patients (18, 19). The receiver operating characteristic (ROC) curve method is used for evaluation of diagnostic performance (20). The area under the curve (AUC) of ROCs of single or multiple factors was calculated using the “ pROC” software package (21). Calibration curves were plotted using the “rms” software package to assess whether the predicted probability of the model approximated the true probability (22, 23).

### Correlation of Diagnostic Markers With Clinical Characteristics

The “ggstatsplot” package was used to perform Spearman correlation analysis of diagnostic markers and clinical features (24), and the results were subsequently visualized using the “ggplot2” package.

### Analysis of the Prognostic Potential of Identified Biomarkers

Kaplan-Meier (KM) curves were constructed using “survival” and “survminer” software packages to assess the probability of DKD occurring at specific time periods, and log-rank tests were used to determine differences between groups (25). The prognostic value of the diagnostic markers was assessed using univariate or multivariate Cox proportional hazards models.

### Clinical Statistics

A total of 141 human blood samples from patients with DKD, DM (without DKD), and healthy individuals, were collected from the biospecimen bank of Shenzhen People’s Hospital. The study was approved by the Ethics Committee of Shenzhen People’s Hospital. Data are expressed as mean plus/minus standard deviation (SD) or median and interquartile range for continuous variables and as percentages for categorical variables. The Mann-Whitney U-test or t-test was performed to compare the differences between the two groups depending on whether the data conformed to a normal distribution. Chi-square test was applied to compare frequencies. This work was approved by the Institutional Review Board and the Ethics Committee of the Shenzhen People’s Hospital (No. LL-KT-2018338) and informed written consent was obtained from all participants.

### Quantitative Real-Time PCR Analysis

Trizol (Invitrogen) was used to extract total RNA from peripheral blood mononuclear cells (PBMC) according to manufacturer’s instructions. Reverse transcription of RNA was performed using the RevertAid RT Reverse Transcription Kit (Thermo Fisher Scientific). Quantitative PCR was performed using PowerUp SYBR Green Master Mix (Thermo Scientific). The results were standardized using GAPDH. qPCR was conducted using an ABI 7500 real-time PCR system (Applied Biosystems, Foster City, CA, USA). Fold-change was determined as 2-^△△Ct^ for gene expression. Gene-specific PCR primers are listed in **Table 1**.

**Table 1:** The sequences of primers for qRT-PCR analysis.

Gene	Forward	Backward
***Homo RUNX3*** **(Gene ID: 864)**	AGGCAATGACGAGAACTACTCC	CGAAGGTCGTTGAACCTGG
**Homo *REG1A*** **(Gene ID: 5967)**	ACCAGCTCATACTTCATGCTGA	CCAGGTCTCACGGTCTTCAT
**Homo *GAPDH*** **(Gene ID: 2597)**	GGAGCGAGATCCCTCCAAAAT	GGCTGTTGTCATACTTCTCATGG

## Results

### Screening for DEGs Shared Across Blood and Kidney Samples

**Figure 2:**
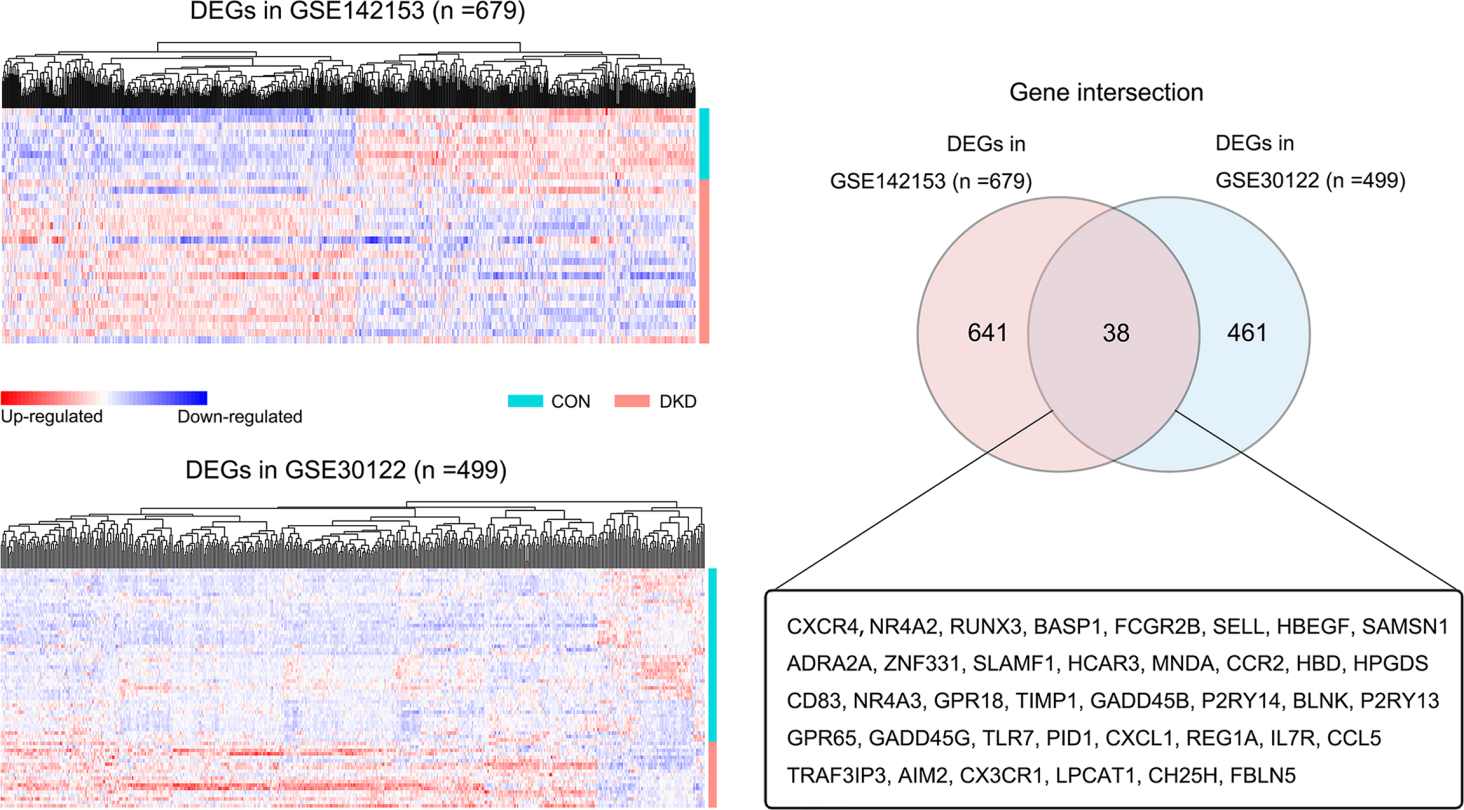
Screening of blood and kidney co-DEGs. Heat map of all DEGs in the blood (GSE142153) and kidney datasets (GSE30122) (DKD vs. HC, p < 0.05). The Venn diagram shows that there are 38 DEGs shared across blood and kidney samples.

**Figure 2** shows the screening process for DEGs shared across blood and kidney biopsy samples from patients with DKD. Using the healthy control samples (HC) as basis, 679 (GSE142153: HC, n=10, DKD, n=23) and 499 (GSE30122: HC, n=50, DKD, n=19) DEGs were identified in blood and kidney samples from patients with DKD, respectively. A total of 38 DEGs were found to be shared across blood and kidney biopsy samples from patients with DKD.

### Screening for DEGs With Consistent Expression in DKD Blood and Kidney Samples

**Figure 3:**
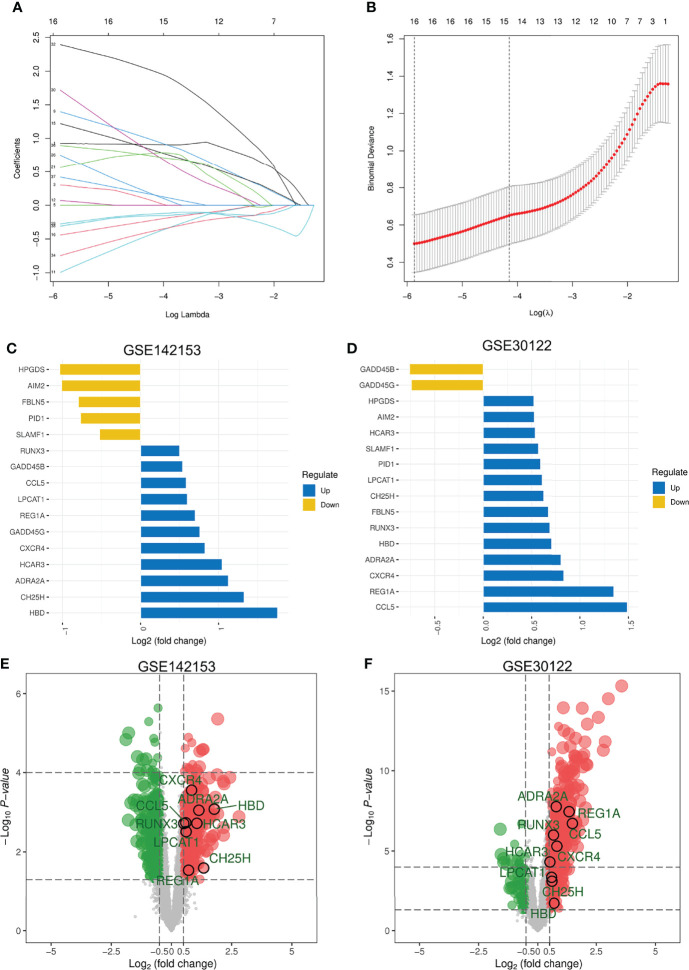
Screening for DEGs with Consistent Expression in DKD Blood and Kidney Samples. **(A)** LASSO coefficient profiles of the 38 features. A coefficient profile plot was produced against the log(λ) sequence. A vertical line was drawn at the value selected using five-fold cross-validation, where the optimal lambda resulted in 16 features with nonzero coefficients. **(B)** Optimal parameter (λ) selection in the LASSO model used five-fold cross-validation *via* minimum criteria. The partial likelihood deviance (binomial deviance) curve was plotted versus log(λ). Dotted vertical lines were drawn at the optimal values by using the minimum criteria and the 1 SE of the minimum criteria (the 1-SE criteria). **(C, D)** Expression of 16 DEGs in blood and kidney. Five DEGs that were consistently expressed in blood and kidney. **(E)** Distribution of these 5 DEGs in DKD blood samples. **(F)** Distribution of the 5 DEGs in the kidney of DKD. LASSO, least absolute shrinkage and selection operator; SE, standard error.

The LASSO logistic regression algorithm was used to further screen DEGs shared across blood samples from patients with DKD (GSE142153). A total of 16 DEGs were identified as diagnostic DKD markers (**Figures 3A, B**), including 5 and 11 down- and up-regulated genes, respectively (**Figure 3C**). Two of these DEGs were down-regulated whereas 14 were upregulated in kidney biopsy samples from patients with DKD (GSE30122) (**Figure 3D**). Ultimately, nine DEGs with consistent expression profiles in blood and kidney biopsy samples from patients with DKD were identified: which included Alpha-2A adrenergic receptor (*ADRA2A*), C-C motif chemokine 5 (*CCL5*), cholesterol 25-hydroxylase (*CH25H*), C-X-C chemokine receptor type 4 (*CXCR4*), hemoglobin subunit delta (*HBD*), hydroxycarboxylic acid receptor 3 (*HCAR3*), lysophosphatidylcholine acyltransferase 1 (*LPCAT1*), Lithostathine-1-alpha (*REG1A*), and Runt-related transcription factor 3 (*RUNX3*). The distribution of the expression profiles of these DEGs in the blood and kidney of DKD patients (**Figures 3E, F**) revealed that expressions of all of them were upregulated.

### Screening for Diagnostic DEGs in DKD

**Figure 4:**
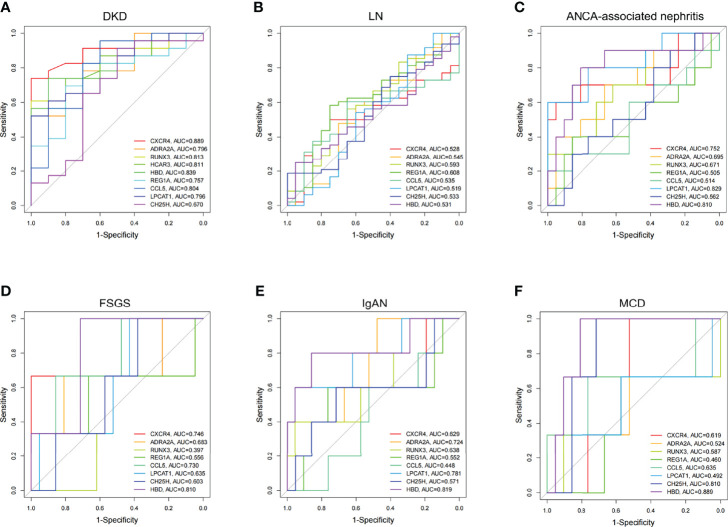
Diagnostic efficacy of DEGs expressed consistently between blood and kidney in CKD. **(A)** Multivariate ROC curve of DEGs in DKD (GSE142153). **(B–F)** Multivariate ROC curve of DEGs in LN, ANCA-associated nephritis, FSGS, IgAN, and MCD, respectively (GSE72326). LN, lupus nephritis; ANCA, Anti-Neutrophil Cytoplasmic Antibodies; FSGS, focal segmental sclerosis; IgAN, IgA nephropathy; MCD, microscopic disease.

Two blood transcriptome datasets were analyzed to determine DKD-specific DEGs for diagnosis. In the GSE142153 dataset, several DEGs (*ADRA2A*, *CCL5*, *CH25H*, *CXCR4*, *HBD*, *HCAR3*, *LPCAT1*, *REG1A*, and *RUNX3*) were found to have high diagnostic efficiency in blood samples (AUC > 0.70), except for *CH25H* (AUC =0.67) (**Figure 4A**). The GSE72326 dataset [lupus nephritis (LN), n=48; ANCA-associated nephritis, n=10; focal segmental sclerosis (FSGS), n = 3; IgA nephropathy (IgAN), n = 5; microscopic disease (MCD), n =3] was used to validate the specificity of these DEGs for DKD diagnosis, except for *HCAR3*, which was excluded from the analysis as it was not found in the dataset. The diagnostic efficacies of eight DEGs in patients with LN were found to be relatively low (**Figure 4B**). *CXCR4*, *LPCAT1*, and *HBD* showed high diagnostic efficacy for ANCA-associated nephritis (**Figure 4C**). Significant diagnostic performance of *CXCR4*, *CCL5*, and *HBD* was observed for patients with FSGS (**Figure 4D**). *ADRA2A*, *LPCAT1*, and *HBD* showed high diagnostic efficacy for patients with IgAN (**Figure 4E**). *CH25H* and *HBD* showed good diagnostic efficacy in patients with MCD (**Figure 4F**). In summary, the transcription level of *REG1A* and *RUNX3* in blood samples may serve as DKD-specific predictors of disease development.

### DKD Diagnosis Efficacy of *RUNX3* and *REG1A* in Blood and Kidney Samples

**Figure 5:**
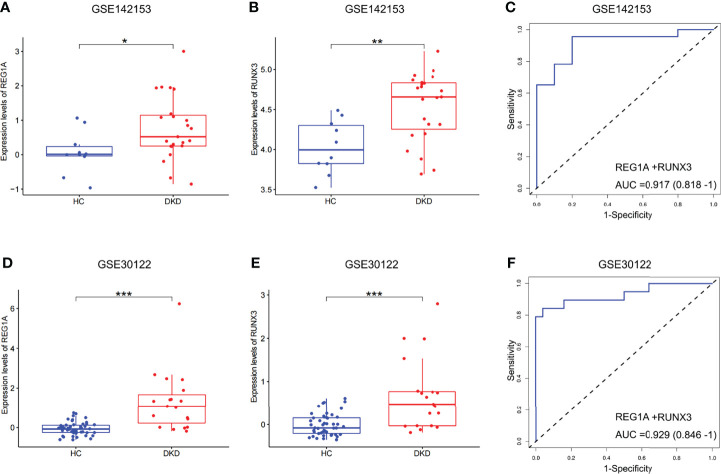
Expression and validation of diagnostic marker efficacy in developmental cohorts. **(A, B)** Box plots showed that the expression levels of *REG1A* and *RUNX3* were significantly higher in blood samples (DKD vs. HC, rank sum test). **(C)** ROC curve of the combined diagnostic efficacy of *REG1A* and *RUNX3* in blood samples. **(D, E)** Box plots showed that the expression levels of *REG1A* and *RUNX3* were significantly higher in the kidney samples (DKD vs. HC). **(F)** ROC curve of the combined diagnostic efficacy of *REG1A* and *RUNX3* in kidney samples. *p <0.05, **p <0.01, ***P <0.001.

Expression levels of *REG1A* and *RUNX3* were found to be significantly increased in blood samples of patients with DKD (**Figures 5A, B**; GSE142153). The combined diagnostic efficacy of these genes was also high (AUC=0.917, 95% CI: 0.818-1) (**Figure 5C**). Similar results have been obtained from kidney samples from DKD patients as well (**Figures 5D, E**; GSE30122, AUC=0.929, 95% CI: 0.846-1, **Figure 5F**). These findings indicate that *REG1A* and *RUNX3* have diagnostic potential for DKD in blood as well as kidney.

### Diagnostic Performance of *REG1A* and *RUNX3* in the Validation Set

**Figure 6:**
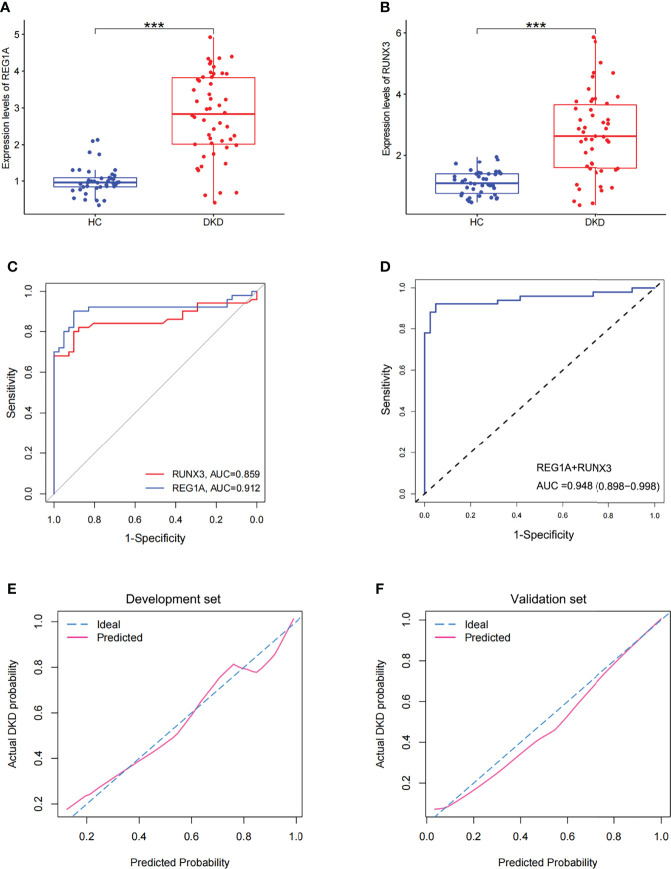
Expression and diagnostic efficacy of diagnostic markers in the validation cohort. **(A, B)** Box plots showed significantly higher expression levels of *REG1A* and *RUNX3* (DKD vs. HC). **(C)** Separate ROC curves of *REG1A* and *RUNX3*. **(D)** The ROC curve of the diagnostic efficacy verification after fitting two diagnostic markers to one variable. **(E, F)** Calibration curves of diagnostic markers. The diagonal dotted line represents a perfect prediction by an ideal model. The solid line represents the performance of the nomogram, of which a closer fit to the diagonal dotted line represents a better prediction. The fit of both the dashed and solid lines for the development set GSE142153 **(E)** and the validation set **(F)** was excellent. ***P <0.001.

A total of 141 blood samples from the human biospecimen bank of Shenzhen People’s Hospital were used for qPCR analysis of *REG1A* and *RUNX3*. DKD (n = 50) and HC (n = 41) groups were included in the validation set (**Table 2**). The expression of *REG1A* and *RUNX3* were found to be significantly upregulated in the DKD group compared to those in the HC group (**Figures 6A, B**). The AUC for *REG1A* and *RUNX3* were found to be 0.912 and 0.859, respectively (**Figure 6C**). When *REG1A* and *RUNX3* were fitted as a single variable, the diagnostic efficiency was found to be 0.917 (**Figure 6C**) for the development set, and even higher for the validation set (AUC=0.948, 95% CI: 0.898-0.998) (**Figure 6D**), indicating that *REG1A* and *RUNX3* have high diagnostic value. A high degree of agreement was also found between the predicted and true values of the calibration curves in both development and validation sets (**Figures 6E, F**), indicating significant efficacy of *REG1A* and *RUNX3* for predicting DKD development.

**Table 2:** Baseline information on diagnostic markers and clinical characteristics in the validation cohort.

	Overall	HC	DKD	p
**n**	91	41	50	
**Female (%)**	32 (35.2)	17 (41.5)	15 (30.0)	0.358
**Age (years)**	56.00 [52.00, 66.00]	54.00 [52.00, 61.00]	57.00 [52.00, 67.00]	0.211
***RUNX3***	1.49 [1.00, 2.82]	1.07 [0.74, 1.38]	2.63 [1.58, 3.66]	<0.001
***REG1A***	1.68 [0.94, 2.98]	0.96 [0.84, 1.09]	2.84 [2.02, 3.82]	<0.001

Data are shown as mean (SD), median [25% quartile, 75% quartile] or numbers (percentages).

### Analysis of Correlation Between Identified DEGs and Clinical Characteristics

**Figure 7:**
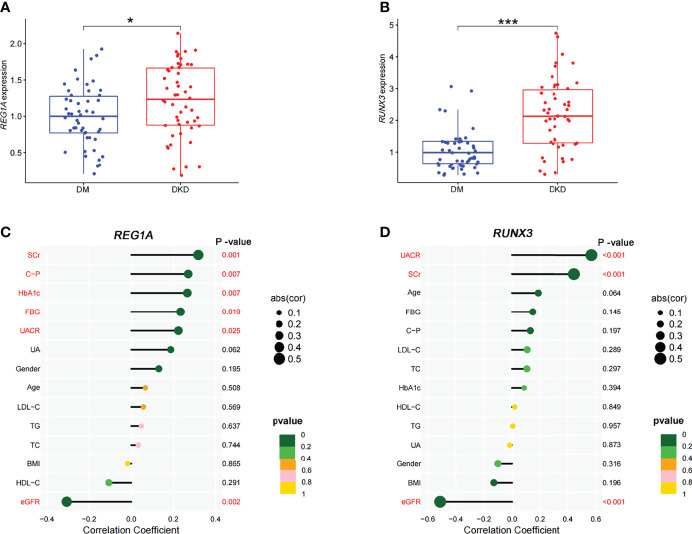
Expression of diagnostic markers and their correlation with clinical characteristics in the DKD and DM cohort. **(A, B)** Box plots showed significantly higher expression levels of *REG1A* and *RUNX3* (DKD vs. DM). **(C)** Correlation of *REG1A* and clinical characteristics. **(D)** Correlation of *RUNX3* and clinical characteristics. The size of the dots represents the correlation between genes and clinical characteristics; the color of the dots represents the p-value. *p <0.05, ***P <0.001.

The DKD (n=50) and DM (without DKD) groups (n=50) in the validation set were used to analyze correlation between expression levels of diagnostic DEGs and clinical characteristics (**Table 3**). Expression levels of *REG1A* and *RUNX3* were found to be significantly higher in the DKD group than those in the DM group (**Figures 7A, B**). *REG1A* was found to be positively correlated with serum creatinine (SCr), C-peptide (C-P), HbA_1C_, fasting blood glucose (FBG), and urinary albumin creatinine ratio (UACR) and negatively correlated with eGFR level (**Figure 7C**, **Supplementary Figure 1**). *RUNX3* was positively correlated with UACR and SCr and negatively correlated with eGFR level (**Figure 7D**, **Supplementary Figure 2**). *REG1A* and *RUNX3* expression levels were found to be positively correlated as well (r=0.3) (**Supplementary Figure 1G**).

**Table 3:** Baseline information on diagnostic markers and clinical characteristics in the DM and DKD groups.

	Overall	DM	DKD	p
**n**	100	50	50	
**Female (%)**	30 (30.0)	15 (30.0)	15 (30.0)	1
**Age (years)**	57.65 (10.49)	56.40 (9.63)	58.90 (11.24)	0.235
**Duration of DM** **(years)**	12.26 (6.59)	10.59 (5.53)	13.56 (7.10)	0.016
**BMI (kg/cm^2^)**	24.10 (3.15)	24.13 (3.15)	24.06 (3.18)	0.916
**FBG (mmol/L)**	6.84 [5.54, 8.51]	6.68 [5.61, 8.44]	7.36 [5.46, 8.48]	0.743
**C-P (ng/ml)**	1.65 [0.89, 2.51]	1.65 [1.24, 2.30]	1.58 [0.67, 3.07]	0.986
**HbA1c (%)**	8.89 (2.09)	8.54 (1.96)	9.25 (2.17)	0.092
**SCr (μmol/L)**	80.50 [65.00, 131.25]	69.50 [60.25, 79.50]	131.50 [86.75, 181.00]	<0.001
**eGFR (ml/min/1.73 m^2^)**	84.21 [47.02, 101.26]	98.13 [88.74, 104.92]	46.30 [32.20, 80.08]	<0.001
**UA (μmol/L)**	375.22 (110.66)	343.68 (97.29)	406.76 (115.08)	0.004
**UACR (mg/g)**	120.12 [7.42, 1932.66]	7.40 [4.27, 16.40]	1951.62[1025.49, 2908.83]	<0.001
**TG (mmol/L)**	1.40 [0.97, 2.16]	1.23 [0.92, 2.13]	1.45 [1.10, 2.17]	0.201
**TC (mmol/L)**	4.53 [3.62, 5.72]	4.28 [3.63, 5.47]	4.85 [3.68, 6.11]	0.222
**HDL-C (mmol/L)**	1.07 [0.89, 1.21]	1.08 [0.93, 1.22]	1.05 [0.82, 1.21]	0.218
**LDL-C (mmol/L)**	2.56 [1.85, 3.73]	2.54 [1.88, 3.45]	2.63 [1.85, 3.77]	0.563
***RUNX3***	1.58 (1.07)	1.00 (0.62)	2.18 (1.10)	<0.001
***REG1A***	1.11 (0.47)	1.00 (0.41)	1.20 (0.49)	0.02

Data are shown as mean (SD), median [25% quartile, 75% quartile], or numbers (percentages). BMI, body mass index; FBG, fasting blood glucose; C-P, C-peptide; HbA1c, glycated hemoglobin A1c; SCr, serum creatinine; eGFR, estimation of glomerular filtration rate; UA, uric acid; UACR, urine albumin creatinine ratio; TG, triglycerides; TC, total cholesterol; HDL-C, high-density lipoproteins cholesterol; LDL-C, low-density lipoproteins cholesterol.

### KM Analysis of Diagnostic Markers and Clinical Characteristics

**Figure 8:**
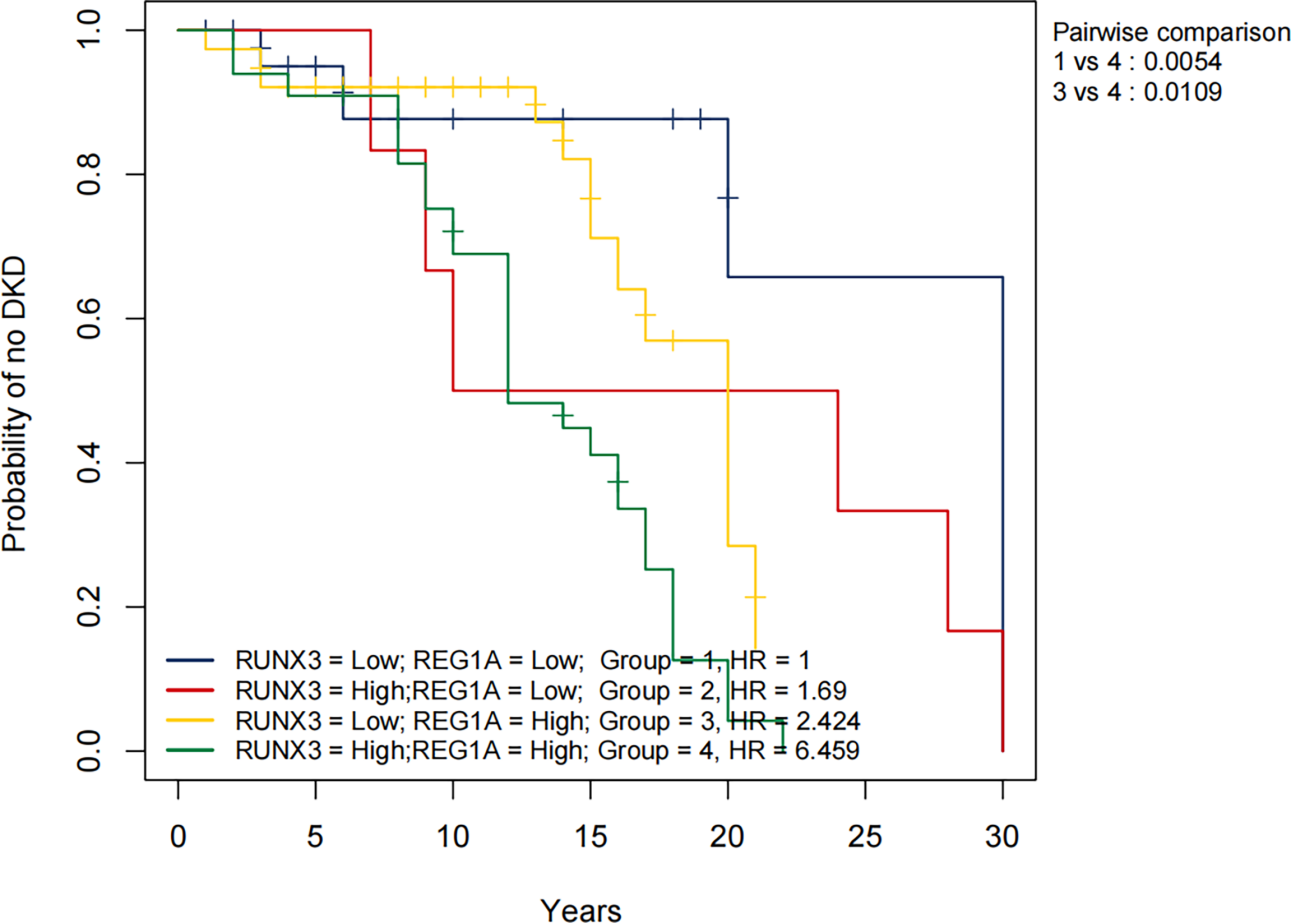
Overall KM curves using combinations of *REG1A* and *RUNX3* expression levels. Univariate Cox regression was used to determine HR; log-rank p-values reported; Bonferroni multiple testing adjustment for pairwise comparisons. *REG1A* high and low cut-offs, 0.84; *RUNX3* high and low cut-offs 1.45. Time unit (years). HR, Hazard ratio.

The KM method was used to analyze the probability of DKD occurrence in the corresponding time periods, and the variables included diagnostic DEGs and clinical characteristics. Using DKD as the endpoint event, variables associated with negative prognosis included *REG1A*, *RUNX3*, total cholesterol (TC), FBG, SCr, body mass index (BMI), and UACR, whereas elevated levels of high-density lipoprotein cholesterol (HDL-C), age, and eGFR indicated a positive prognosis (**Table 4** and **Supplementary Figure 3**). Combined analysis showed that patients with high expression of *REG1A* and *RUNX3* had the worst prognosis in all four groups (HR = 6.459) (**Figure 8**).

**Table 4:** Univariate COX regression of diagnostic markers and clinical characteristics.

Variable	HR	lower 95%CI	upper 95%CI	p-value
*RUNX3*	2.50	1.43	4.36	<0.001
*REG1A*	2.97	1.35	4.17	0.003
TC	1.93	1.11	3.39	0.022
FBG	1.78	1.01	3.12	0.029
SCr	2.44	1.40	4.25	0.001
HDL-C	0.51	0.29	1.02	0.022
Age	0.4	0.22	0.75	<0.001
eGFR	0.34	0.19	0.61	0.002
BMI	1.84	1.02	3.31	0.019
UACR	5.62	3.22	9.81	<0.001

BMI, body mass index; FBG, fasting blood glucose; SCr, serum creatinine; eGFR, estimation of glomerular filtration rate; UACR, urine albumin creatinine ratio; TC, total cholesterol; HDL-C, high-density lipoproteins cholesterol. Showing variables with p-values <0.05.

## Discussion

In the present study, we found that serum RUNX3 and REG1A have potential as diagnostic markers for DKD. Patients with DM who have elevated expression of both RUNX3 and REG1A will have a much higher risk of developing DKD at about 10 years into the disease. Compared to existing studies of diagnostic markers for DKD (26, 27), we have achieved high diagnostic efficacy with the combination of only two markers. Importantly, the diagnostic efficacy of RUNX3 and REG1A in blood is not inferior to that of renal tissue, which facilitates their widespread use. RUNX3 and REG1A are more specific in the diagnosis of DKD compared to markers such as urinary transferrin, urinary IgG and urinary type IV collagen (28).

RUNX transcription factors regulate various biological processes, including embryonic development, cell proliferation, differentiation, cell lineage determination, and apoptosis (29). *RUNX3* plays a downstream role in the TGF-β signalling pathway (30). Smyth et al. reported that *RUNX3* methylation is significantly increased in the blood of patients with DKD (31). In high glucose-treated renal tubular epithelial cells, RUNX3 was found to regulate the TGF-β1/Smad signalling pathway (32). Inhibition of *Runx3* expression ameliorates vascular endothelial dysfunction in DM mice (33). In diabetic patients, *RUNX3* was similarly found to be involved in diabetic endothelial progenitor cell dysfunction (34). High expression of *RUNX3* may exacerbate diabetic vascular endotheliopathy and ultimately lead to DKD. The REG family proteins are structurally similar to each other, and classified as calcium-dependent lectins (35). The mainstream view is that *REG1A* is an indicator of islet β-cell apoptosis and its elevation indicates a decline in islet function (36). In contrast, Okamoto et al. found that *REG1A* stimulates islet regeneration by inducing cell proliferation (37). Sobajima et al. reported significantly increased urinary *REG1A* levels in patients with DKD (38). Li et al. found that REG1A levels are significantly upregulated in the serum of patients with DKD, and possibly associated with renal injury as well (39). The above suggests that REG1A level is associated with impairment of islet function and DKD renal function. The above suggests that REG1A level is associated with islet and DKD renal injury. Our study also found that serum *REG1A* was significantly and positively associated with renal and islet impairment, while serum *RUNX3* was only associated with renal impairment. Our study also found that serum *REG1A* was significantly elevated in DM, whereas *RUNX3* was unchanged (**Supplementary Figure 4**). We therefore speculate that *REG1A* causes diabetes by destroying islet cells, whereas *RUNX3* causes DKD by directly destroying vascular endothelial cells.

To assess the risk of DKD development, we plotted KM curves for *REG1A*, *RUNX3* and the clinical characteristics. The results showed that people with high *REG1A* and *RUNX3* expression are at an increased risk of DKD after approximately 12 and 8 years of DM, respectively. Our findings are consistent with the results of most previous studies (40–44), which revealed incresaed TC, FBG, SCr, BMI, and UACR levels as risk factors for DKD, and increased eGFR and HDL-C levels are protective factors for DKD. Contrary to previous findings (45), aging was found to be a protective factor against DKD in this study. Afkarian et al. reported that youth-onset type 2 DM patients may have a higher risk of DKD (46). **Figure 8** shows the KM curves for different expression levels of *REG1A* and *RUNX3*. Accordingly, the risk of DKD was found to be highest when expression levels of both *REG1A* and *RUNX3* are high compared to other groups. Compared to patients with low expression of both *REG1A* and *RUNX3*, DM patients with high expression of both genes are at a rapidly increasing risk of developing DKD after 7-8 years of DM. Hence, *REG1A* and *RUNX3* are potential biomarkers for predicting the risk of developing DKD.

### Limitation

In this study, when comparing the diagnostic efficacy of DEGs in chronic kidney disease (GSE72326), insufficient sample size may have led to unreliable results. We plan to continue collecting blood from patients with chronic kidney disease in subsequent studies to determine the specificity of *RUNX3* and *REG1A* in the diagnosis of DKD. We have so far only found elevated *RUNX3* and *REG1A* at the transcriptional level in the kidney and subsequent collection of kidney tissue from DKD patients for immunohistochemistry and protein immunoblotting is required.

### Conclusion

*REG1A* and *RUNX3* were found to have high diagnostic efficacy for DK, which was proven through external validation. *REG1A* and *RUNX3* levels were positively and negatively correlated with UACR and eGFR levels, respectively. Thus, the transcription levels of *REG1A* and *RUNX3* in blood samples have potential to predict DKD risk.

## Data Availability Statement

The original contributions presented in the study are included in the article/**Supplementary Material**. Further inquiries can be directed to the corresponding authors.

## Ethics Statement

The studies involving human participants were reviewed and approved by Shenzhen people’s hospital. Written informed consent for participation was not required for this study in accordance with the national legislation and the institutional requirements.

## Author Contributions

XW, HW and GY contributed equally to this work. XW, GY, HW and JX contributed to the acquisition of data, analysis and interpretation of data. SY and XW drafted the work or revised it critically for important intellectual content. LX, LZ, TL, and XZ, and LK analyzed the data and revised the article critically for important intellectual content. ZL and SY contributed to the conception and the study design. All authors gave their approval of the version to be published. All authors contributed to the article and approved the submitted version.

## Funding

This work was supported by grants from the National Key Research and Development Program of China (2018YFC2001100); the National Natural Science Foundation of China (No. 82000824 to SY, No. 82171556 to LK, and No. 82170842 to ZL).

## Conflict of Interest

The authors declare that the research was conducted in the absence of any commercial or financial relationships that could be construed as a potential conflict of interest.

## Publisher’s Note

All claims expressed in this article are solely those of the authors and do not necessarily represent those of their affiliated organizations, or those of the publisher, the editors and the reviewers. Any product that may be evaluated in this article, or claim that may be made by its manufacturer, is not guaranteed or endorsed by the publisher.
